# Cytokine-based immunotherapy for gastric cancer: targeting inflammation for tumor control

**DOI:** 10.37349/etat.2025.1002312

**Published:** 2025-04-26

**Authors:** Mathan Muthu Chinakannu Marimuthu, Bhavani Sowndharya Balamurugan, Vickram Agaram Sundaram, Saravanan Anbalagan, Hitesh Chopra

**Affiliations:** IRCCS Istituto Romagnolo per lo Studio dei Tumori (IRST) “Dino Amadori”, Italy; ^1^Department of Biotechnology, Saveetha School of Engineering, Saveetha Institute of Medical and Technical Sciences, Chennai 602105, Tamil Nadu, India; ^2^Centre for Research Impact & Outcome, Chitkara College of Pharmacy, Chitkara University, Rajpura 140401, Punjab, India

**Keywords:** Cytokine-based immunotherapy, gastric cancer, inflammation, interleukin-2 (IL-2), interferons (IFNs), tumor necrosis factor-alpha (TNF-α), tumor microenvironment (TME)

## Abstract

Emerging cancer immunotherapy methods, notably cytokine-based ones that modify immune systems’ inflammatory reactions to tumor cells, may help slow gastric cancer progression. Cytokines, tiny signaling proteins that communicate between immune cells, may help or hinder cancer growth. Pro-inflammatory cytokines encourage tumor development, whereas antitumor ones help the host reject cancer cells. This study considers cytokine-targeted methods for gastric cancer pro-inflammatory and antitumor immune responses. Researchers want to renew immune cells like cytotoxic T lymphocytes (CTLs) and natural killer (NK) cells by delivering cytokines like interleukin-2 (IL-2), interferons (IFNs), and tumor necrosis factor-alpha (TNF-α) to activate inflammatory pathways and combat tumors. Since cytokines have significant pleiotropic effects, their therapeutic use is difficult and may cause excessive systemic inflammation or immunological suppression. This review covers current advancements in synthetic cytokines, cytokine-conjugates, and local administration of these aimed to enhance the therapeutic index: increase the potential to kill cancer cells while minimizing off-target damage. The study examines the relationship between cytokines and tumor microenvironment (TME), revealing the role of immunosuppressive cytokines like IL-10 and transforming growth factor-beta (TGF-β) in promoting an immune-evasive phenotype. These results suggest that inhibitory pathway targeting, and cytokine-based therapy may overcome resistance mechanisms. Cytokine-based immunotherapies combined with immune checkpoint inhibitors are predicted to change gastric cancer therapy and rebuild tumor-immune microenvironment dynamics, restoring antitumor immunity. Comprehensive data from current clinical studies will assist in establishing the position of these treatments in gastric cancer.

## Introduction

Despite tremendous breakthroughs in diagnostic and therapeutic procedures, cancer remains one of the world’s most serious health problems, with rising incidence and fatality rates. Gastric cancer, for example, is one of the most common and lethal diseases in the world, posing significant challenges to early detection and treatment [1]. Its diverse pathophysiology is characterized by a complex combination of genetic alterations, environmental stressors, and immune system dysregulation, demanding research into novel therapeutic approaches. The immune system’s role in modulating cancer formation and progression has received a lot of attention, particularly the significance of cytokines, which are tiny, secreted proteins that are crucial for immune control and signaling [2]. Cytokines are essential for maintaining immunological homeostasis and regulating immune responses, acting as mediators during inflammation, cell differentiation, and immune and non-immune cell communication. Cytokines play a dual role in cancer biology: depending on the context and the cytokine profile inside the tumor microenvironment (TME), they can either enhance antitumor immunity or promote tumor growth and immune evasion [3]. The TME is a complex and dynamic milieu that includes cancer cells, stromal cells, immunological infiltrates, and signaling molecules, with cytokines influencing tumor behavior and therapeutic responses. Cytokines’ role in gastric cancer has emerged as an important field of research due to their major influence on tumor suppression and development. Pro-inflammatory cytokines including interleukin-1 (IL-1), IL-6, and tumor necrosis factor-alpha (TNF-α) promote inflammation-induced carcinogenesis while also stimulating antitumor immune responses [4]. Immunosuppressive cytokines such as IL-10 and transforming growth factor-beta (TGF-β) create a milieu that hinders antitumor activity, leading to immune evasion [5, 6]. Understanding the balance of these cytokines is critical for the effective execution of immunotherapeutic therapies.

Cytokines have proven to be an effective tool in gastric cancer immunotherapy due to their ability to activate immune effector activities and modify the TME. IL-2 was one of the first cytokines used in cancer immunotherapy, and it demonstrated clear antitumor activity by increasing T cell proliferation and activation [7, 8]. Interferons (IFNs) have also played an important role in boosting antitumor immunity due to their antiviral and immunomodulatory properties. Despite their therapeutic promise, these cytokines are known to cause systemic toxicity and have little efficacy when administered alone. Cytokine therapeutics have advanced to overcome these restrictions through the use of tailored cytokines, cytokine conjugates, and local delivery systems [9]. Engineered cytokines with enhanced pharmacokinetics and fewer side effects offer a promising technique for enhancing antitumor responses while minimizing toxicity. Targeted cytokine conjugates can modulate TME-specific immune responses, increasing treatment efficacy and safety. Localized cytokine delivery systems further reduce systemic exposure by increasing cytokine activity at the tumor site, which ensures therapy specificity [10]. Combining cytokine-based therapy with other treatments has resulted in synergistic benefits, paving the path for more beneficial combinations. The combination of immune checkpoint inhibitors (ICIs) and cytokines has shown promise in overcoming immune resistance and promoting antitumor immunity. In a similar vein, combining cytokine therapy with chemotherapy and radiotherapy has improved therapeutic outcomes by modifying the immune response and making tumors more vulnerable to normal therapies [11, 12]. These combinatorial regimens offer a comprehensive strategy for addressing the poly-facetic nature of stomach cancer. Despite these accomplishments, a few difficulties remain in the way of the clinical application of cytokine-based immunotherapy. Management of cytokine-related toxicity and side effects is critical, and measures must be developed to mitigate negative consequences while maintaining therapeutic efficacy [13]. The creation of consistent biomarkers for predicting response to cytokine therapy is an ongoing research topic with the potential to improve patient selection and individualized treatment. Overcoming immune resistance through cytokine regulation requires better knowledge of the TME’s complex interconnections [14].

New cytokine treatments and tailored medicine tactics show great promise for the future of gastric cancer treatment. Advances in genetic profiling, biomarker identification, and precision medicine have the ability to tailor cytokine-based treatments to individual patient profiles, increasing therapeutic efficacy while lowering toxicities [15, 16]. With continuous research elucidating the complexity of cytokine signaling in cancer, the development of new cytokine-based techniques is poised to revolutionize gastric cancer treatment. This article aims to provide an in-depth analysis of the role of cytokines in gastric cancer, including immune modulation, tumor suppression, and immune evasion. This study demonstrates the revolutionary potential of cytokines in shaping the future of gastric cancer immunotherapy by reviewing existing and future cytokine-based medicines, combination therapy, and directions.

## Cytokines in gastric cancer

Cytokines have a double-edged sword in the immune system as they are important modulators of inflammation and neoplasia development, involving gastric carcinoma among themselves. This part therefore discusses various ways through which pro-inflammatory and immunosuppressive cytokines play important roles in cancer regression and escape from host immunity respectively; it also examines how these different kinds of cytokines interact with one another within TME affecting disease prognosis. Different cytokines and their roles in gastric cancer in depicted in Table 1.

**Table 1:** Cytokines and their roles in gastric cancer

**Sl. No.**	**Cytokine**	**Type**	**Role in gastric cancer**	**Mechanism**	**Potential therapeutic target**	**Reference**
1	IL-2	Pro-inflammatory	Tumor suppression	Stimulates T cells and NK cells	Cancer immunotherapy	[17]
2	IFN-α	Pro-inflammatory	Enhances antitumor immunity	Activates immune responses	Cytokine therapy	[18]
3	TNF-α	Pro-inflammatory	Tumor necrosis	Induces apoptosis in tumor cells	Localized cytokine delivery	[19]
4	IL-10	Immunosuppressive	Promotes tumor evasion	Suppresses immune system	Anti-IL-10 antibodies	[20]
5	TGF-β	Immunosuppressive	Promotes tumor growth and metastasis	Modulates T cell response	TGF-β inhibitors	[21]
6	IL-6	Pro-inflammatory	Tumor progression	Activates signaling pathways in cancer cells	Anti-IL-6 therapy	[22]
7	IL-8	Pro-inflammatory	Promotes angiogenesis and metastasis	Attracts immune cells to tumor sites	CXCR2 inhibitors	[23]
8	IL-1β	Pro-inflammatory	Tumor proliferation	Activates inflammatory pathways	IL-1 receptor antagonists	[24]
9	*IL-12*	Pro-inflammatory	Stimulates cytotoxic T cells	Induces Th1 responses	*IL-12* agonists	[22]
10	IL-17	Pro-inflammatory	Tumor growth and angiogenesis	Activate pro-inflammatory pathways	Anti-IL-17 antibodies	[17]
11	IL-22	Immunosuppressive	Tumor promotion	Modulates immune responses in TME	IL-22 inhibitors	[25]
12	GM-CSF	Pro-inflammatory	Enhances tumor immune response	Stimulates dendritic cells	GM-CSF cytokine therapy	[26]
13	IL-15	Pro-inflammatory	Enhances T and NK cell activity	Increasing cytotoxic activity	IL-15 superagonists	[27]
14	IFN-γ	Pro-inflammatory	Antitumor immune response	Activates macrophages and CTLs	IFN-γ therapy	[28]
15	IL-18	Pro-inflammatory	Promotes tumor immunity	Induces IFN-γ production	IL-18 agonists	[29]

IL-2: interleukin-2; NK: natural killer; IFN-α: interferon-alpha; TNF-α: tumor necrosis factor-alpha; TGF-β: transforming growth factor-beta; TME: tumor microenvironment; CTLs: cytotoxic T lymphocytes

### Pro-inflammatory cytokines and tumor suppression

Pro-inflammatory cytokines appear to play a crucial role in the orchestration of antitumor immune responses, as they activate the effector cells for the recognition and killing of tumor cells. The roles that such cytokines, such as IL-2, TNF-α, and IFNs, could play would prove to be of significant importance in the suppression of gastric cancers.

IL-2 is highly efficient in stimulating the proliferation and activation of cytotoxic T lymphocytes (CTLs) and natural killer (NK) cells, which are important effectors in antitumor immunity. Various clinical studies have also indicated that IL-2 administration generates immune-mediated tumor regression in patients with gastric cancers. However, systemic administration of IL-2 often results in severe toxicities due to its pleiotropic effects. Hence, there has been the development of engineered IL-2 variants aimed at improving the therapeutic index while reducing side effects [30].

Another key pro-inflammatory cytokine is TNF-α, which, through the activation of caspase pathways, causes cell death among the tumor cells but also induces the infiltration of immune cells at the site of the tumor, thus enhancing local antitumor immunity. The dual role of TNF-α, in both tumor growth enhancement and suppression complicates its use for therapy. Chronic inflammation caused by excessive TNF-α leads to a tumor-supportive rather than a tumor-suppressive environment [31].

IFNs, especially IFN-γ, are found to be crucial for the induction of antitumor immune responses, mainly through promoting the functioning of antigen presentation and the activation of NK cells and CTLs. Also, IFN-γ can suppress tumor cell proliferation by triggering cell cycle arrest and apoptosis. So far, clinical applications of IFN-based therapies in gastric cancer are still being investigated, but some early results suggest a potential role in combination with other types of immune-modulating therapy [32].

### Immunosuppressive cytokines and tumor evasion

Although pro-inflammatory cytokines help in controlling tumor growth, the tumor can avoid immunity with immunosuppressive cytokines like IL-10 and TGF-β, which suppress the immune system.

IL-10 is a very strong anti-inflammatory cytokine that anergizes macrophages and dendritic cells thereby reducing the ability of the immune system to detect and destroy tumor cells. High levels of IL-10 in gastric cancer patients are associated with poor prognosis since it supports a tolerogenic environment for the tumor to evade immune surveillance [33]. The role of IL-10 in increasing regulatory T cell (Treg) population further aids in immune suppression that can dampen effective antitumor responses.

TGF-β is another important immunosuppressive cytokine that promotes tumor progression through the induction of epithelial-mesenchymal transition, whereby tumor cells acquire their invasive and metastatic potential. TGF-β inhibits the activity of effector T cells and NK cells further impairing the host’s immune defense against tumor growth. In gastric cancer, high levels of TGF-β correlate with more advanced disease stages and metastasis [34].

### Cytokine networks in TME

TME cytokine networks are a highly complex and dynamic system in which the interplay of immune cells, stromal components, and signaling molecules controls tumor progression and immune escape. The ratio of pro-inflammatory to immunosuppressive cytokines is an important regulator of the immunological milieu in the TME, influencing both antitumor responses and tumor-supportive pathways. Pro-inflammatory cytokines like IL-2, IFN-γ, and TNF-α play crucial roles in promoting antitumor immunity. IL-2 stimulates the development and activation of CTLs and NK cells, which are essential for detecting and eliminating tumor cells. IFN-γ enhances antigen presentation by increasing major histocompatibility complex (MHC) molecules on tumor cells, making them more apparent to immune effectors. TNF-α activates caspase pathways, causing death in tumor cells and attracting immune cells to the tumor, leading to a localized antitumor response. Immunosuppressive cytokines such as IL-10 and TGF-β create an immune-evasive milieu, promoting tumor survival and proliferation. IL-10 inhibits the activation of dendritic cells and macrophages, reducing antigen presentation and inhibiting effector T cell responses. It also promotes Treg expansion, which inhibits antitumor immunity. TGF-β promotes EMT, which increases tumor invasiveness and metastatic potential. TGF-β suppresses CTL and NK cells, promoting Treg development and creating an immunosuppressive environment within the TME. This reciprocal interaction between cytokines dynamically shapes the immunological environment of the TME. The tumor-associated macrophage (TAM) is a paradigmatic example, with different morphologies regulated by cytokine signals. M1-polarized TAMs produce pro-inflammatory cytokines such as IL-12 and TNF-α, which increase antitumor action. M2-polarized TAMs release IL-10 and TGF-β, which promote immune suppression and tumor development. New treatment techniques aim to alter cytokine networks and shift the TME from immunosuppressive to immunostimulatory. Engineered cytokines, cytokine conjugates, and local delivery systems are being developed to improve the therapeutic efficacy of cytokine-based treatments. Cytokine treatment paired with ICIs has shown promise in breaking immunological resistance, rejuvenating tired T cells, and restoring robust antitumor immunity. Improved understanding of cytokine networks inside the TME is required to design tailored therapies that disrupt immunosuppressive pathways and boost antitumor immunity. To maximize the success of cytokine-based therapeutics, future studies will look for biomarkers that predict treatment response, as well as tailored techniques. Pro-tumor cytokines like IL-10 and TGF-β promote an immune-evasive environment, while antitumor cytokines like IL-2 and TNF-α strive to elicit immune-mediated tumor destruction. Targeting these cytokine networks to shift the TME from an immunosuppressive state to an immunostimulatory one is a promising therapeutic strategy for improving patient outcomes in gastric cancer [35].

### Cytokines for gastric cancer immunotherapy

Gastric cancer continues to be one of the prominent causes of mortality from cancer around the globe, and cytokine immunotherapy holds the promise as an effective way to increase cancer treatment. Given that cytokines are essential mediators of immunity, they have a dual purpose—either to promote antitumor immunity or allow for immune suppression by tumors. Unleashing the therapeutic potential of cytokines involves a detailed grasp of their processes and the challenges with their clinical application. Current development is geared toward maximizing cytokine-based therapy, maximizing its efficacy while decreasing systemic toxicity [36].

#### IL-2: mechanisms of action and therapeutic potential

IL-2 has long been a cornerstone in cytokine-based immunotherapy because of its significant capability for boosting the activation and proliferation of CTLs and NK cells. Through amplification of these effector cells, IL-2 enhances the detection and destruction of stomach cancer cells. In spite of its potential, the therapeutic use of IL-2 is limited by acute toxicities, including vascular leak syndrome and systemic inflammation, stemming from its pleiotropy. New breakthroughs have given rise to tailored variants of IL-2 that have been tuned to preferentially activate effector cells without associating heavily with Tregs, in turn decreasing undesirable effects. Furthermore, localized delivery techniques and IL-2 conjugates are being studied to restrict cytokine activity to the TME, which would increase therapeutic efficacy and safety [37]. New tactics for IL-2 therapy also aim at dose optimization and combination therapy to reduce adverse effects and maximize antitumor results. Low-dose IL-2 regimens have been promising to boost Treg populations and immunomodulate immunological balance to generate a better environment for persistent antitumor activity. In addition, innovative delivery techniques, including nanoparticle-encapsulated IL-2, are being studied to maximize targeted delivery and limit systemic exposure. These discoveries underscore the promise of IL-2 in rethinking gastric cancer therapy by harnessing its powerful immunostimulatory action with lower safety hazards.

#### IFNs: regulation of antitumor immunity

IFNs, notably IFN-α and IFN-γ, are pivotal in regulating antitumor immune responses by increasing antigen presentation and activating immune effector cells. IFN-γ promotes tumor cell production of MHC molecules, boosting their visibility to CTLs and improving immune-mediated tumor lysis. IFNs have also demonstrated promise as immunotherapies for gastric cancer, especially as combination treatments with other immune-modulating drugs. Their use as clinical drugs, meanwhile, has been restricted because of adverse effects such as flu-like illness, lethargy, and depression [38]. Recent research aims IFN delivery optimization with targeted administration and synergistic combos with ICIs to obtain maximal antitumor effects with minimal systemic damage. Aside from their antitumor direct activities, IFNs also affect TME by modifying the behavior of stromal and immune cells. For example, IFN-γ can activate macrophages and dendritic cells to improve their antigen-presentation capacities and form a more immunogenic TME. Clinical experiments studying the administration of IFNs in combination with adoptive T cell therapies and cancer vaccines have shown promising outcomes, which indicate the possibility of synergistic effects. Investigations of modifying IFN variants with longer half-lives and reduced toxicity further underline the continuous interest in preserving efficacy while assuring safety in IFN therapy. These various capabilities place IFNs in a pivotal role in developing cytokine-based immunotherapy for gastric cancer.

#### TNF-α: balancing efficacy and toxicity

TNF-α demonstrates a multifaceted role in gastric cancer immunotherapy, operating as both a tumor suppressor and a promoter depending on its position within the TME. At high quantities, TNF-α promotes death in tumor cells and attracts immune cells to the tumor site, enhancing local immune responses. Conversely, prolonged low-level TNF-α expression can accelerate inflammation-induced tumor development. The systemic usage of TNF-α is constrained by substantial toxicities, like fever, hypotension, and tissue injury. New ideas, such as targeted TNF-α delivery systems and TNF-α conjugates developed by engineering, strive to localize cytokine activity while limiting off-target effects without compromising antitumor efficacy. Studies involving TNF-α have also focused on harnessing its dual function through context-dependent usage. In conjunction with radiotherapy and chemotherapy, TNF-α improves tumor cell sensitivity to apoptosis produced by therapy, boosting overall therapeutic response. In addition, *TNF-α* gene treatments and oncolytic viral techniques are also researched for the ability to give localized cytokine action directly within the TME [39]. The discovery of protein engineering has developed TNF-α fusion proteins with better tumor specificity and lower systemic toxicity, presenting new options for more effective, safer treatment. These cutting-edge tactics match the dynamic nature of TNF-α therapy for gastric cancer, balancing the potent anti-cancer effects with the necessity of management and intentional administration. In conclusion, cytokine-based treatments are promising approaches for gastric cancer. Improved synthetic cytokines, tailored delivery systems, and combination therapy with ICIs are leading the way toward safer and more successful treatment strategies. Continued research must concentrate on balancing the efficacy of cytokines with minimizing systemic toxicity in order to offer the best patient results.

## Cytokine-based therapies in clinical application

Immunotherapies based on cytokines hold a lot of promises for the treatment of gastric cancer because such an approach modulates the host immune response directly at the site of the tumor. However, the pleiotropic nature of cytokines limits their therapeutic usage in the clinic due to the accompanying systemic toxicity [40]. For such reasons, engineered cytokines and cytokine-conjugates, as well as delivery systems localized to the tumor site, have been developed to achieve enhanced efficacy with decreased toxicity. Table 2 provides an overview of these innovations in clinical implications of such undertakings in gastric cancer.

**Table 2:** Cytokine-based therapies in clinical application

**Sl. No.**	**Therapy**	**Type**	**Mode of action**	**Challenges**	**Clinical trials**	**Reference**
1	Engineered IL-2	Pro-inflammatory	Enhanced activation of T and NK cells	Systemic toxicity	Phase I/II	[41]
2	Pegylated IFN-α	Pro-inflammatory	Prolonged immune activation	Limited efficacy in solid tumors	Ongoing	[42]
3	TNF-α conjugates	Pro-inflammatory	Targeted tumor necrosis	Off-target effects	Preclinical	[43]
4	Anti-IL-10 antibodies	Immunosuppressive	Inhibits immunosuppressive IL-10	Immune-related adverse events	Phase I	[44]
5	TGF-β inhibitors	Immunosuppressive	Blocks TGF-β signaling in TME	Off-target inhibition	Phase II	[45]
6	IL-6R antagonists	Pro-inflammatory	Reduces tumor-associated inflammation	Potential for autoimmune reactions	Phase II	[46]
7	*IL-12* gene therapy	Pro-inflammatory	Induces strong antitumor immune responses	Delivery challenges	Preclinical	[47]
8	IFN-γ therapy	Pro-inflammatory	Enhances macrophage activation	Short half-life	Ongoing	[48]
9	GM-CSF-based vaccines	Pro-inflammatory	Activates dendritic cells for immune priming	Inconsistent immune responses	Phase I	[49]
10	IL-15 superagonists	Pro-inflammatory	Amplifies T and NK cell cytotoxicity	Cytokine release syndrome	Phase I/II	[50]
11	IL-22 inhibitors	Immunosuppressive	Reduces immune evasion in TME	Specificity issues	Preclinical	[51]
12	Combination of IL-2 and checkpoint inhibitors	Pro-inflammatory	Synergizes T cell activation	Severe toxicity risks	Phase III	[52]
13	Pegylated IL-18	Pro-inflammatory	Enhances IFN-γ production	Uncertain dosing	Phase I	[53]
14	Localized TNF-α delivery	Pro-inflammatory	Targets tumor directly	Local inflammation	Preclinical	[54]
15	IL-1 receptor antagonists	Pro-inflammatory	Blocks IL-1-mediated tumor growth	Potential immune suppression	Ongoing	[55]

IL-2: interleukin-2; NK: natural killer; IFN-α: interferon-alpha; TNF-α: tumor necrosis factor-alpha; TGF-β: transforming growth factor-beta; TME: tumor microenvironment

### Engineered cytokines for enhanced antitumor responses

Engineered cytokines are recombinantly engineered to retain the desired characteristics of their natural counterparts while being much more specific with minimal off-target effects. For example, modified variants of IL-2 have been designed to selectively engage effector T cells over Tregs that suppress the natural antitumor immune response. Alkermes (ALKS) 4230 represents one such engineered cytokine that engages and expands the antitumor activities of effector T cells and NK cells, potentially leading to improved clinical outcomes in gastric cancer patients [56].

More significantly, new developments in protein engineering have been established. For example, long-acting cytokine formulations, such as PEGylated cytokines, express a longer half-life and reduce toxicity. These new formulations ensure continued cytokine activity at the site of the tumor, thus enhancing the therapeutic response with reduced frequencies of administration [57]. Early clinical trials using engineered cytokines have shown promising results, documenting increased antitumor activity but with fewer side effects among patients with different malignancies, including gastric malignancy.

### Cytokine-conjugates: targeted delivery for improved safety

Another innovative means of improving the safety of cytokine-based therapies is through cytokine-conjugates. Cytokine-conjugates consist of conjugating cytokines with targeting molecules, including antibodies or peptides, that locate cytokine activity to the TME. Therefore, localized delivery of cytokines to the TME by cytokine-conjugates leads to lesser systemic exposure and decreases off-target effects such as immune-related toxicities [58].

For instance, the IL-2 conjugated with tumor-targeting antibodies showed promise in the preclinical models of gastric carcinoma. These conjugates selectively deliver IL-2 to the target sites of the tumor, allowing for the activation of the antitumor immune cells without causing activation of the normal tissues [59]. The targeted approach is not only beneficial in improving the therapeutic index but also allows for higher doses of cytokines with reduced toxicity.

### Localized cytokine delivery systems: reducing systemic toxicity

Localized cytokine delivery systems have been developed to address the systemic toxicity associated with cytokine therapy by limiting the activity of cytokines at the tumor site. The delivery systems employed are diverse and range from hydrogel-based systems to nanoparticle-encapsulated delivery systems. Such delivery systems, once stabilized in the body, would be available for controlled cytokine delivery, ensuring the therapeutic concentrations at the tumor site and minimizing systemic exposure.

For instance, hydrogels loaded with IL-12 were shown to hold promise in preclinical models of gastric cancer through sustained local release of cytokines [60]. Such delivery systems increase localized immunity and trigger tumor regressions without the severe adverse effects of systemic cytokine therapies. Targeted delivery of cytokines through nanoparticle-encapsulated cytokines seems to serve as an effective alternative approach to precision therapy for gastric cancer [61, 62].

## Combination therapies with cytokines

Cytokine-based immunotherapies are increasingly being used for the management of cancers, particularly gastric cancer. In many cases, however, cytokine therapies alone have limitations and may not be most effective. The integration of cytokine therapies along with treatment modalities such as ICIs, chemotherapy, and radiotherapy can have a higher probability of positive outcomes as shown in Table 3 since a multifactor mechanism in the cancer could be targeted.

**Table 3:** Combination therapies with cytokines

**Sl. No.**	**Combination therapy**	**Cytokine used**	**Other therapy**	**Synergistic effect**	**Challenges**	**Reference**
1	IL-2 and immune checkpoint inhibitors	IL-2	PD-1/PD-L1 inhibitors	Enhance T cell activation	Severe immune-related toxicity	[63]
2	IFN-α with chemotherapy	IFN-α	Doxorubicin	Potentiates chemotherapy-induced tumor killing	Limited duration of response	[64]
3	TNF-α and radiotherapy	TNF-α	Localized radiation	Synergizes with radiation-induced cell death	Local tissue damage	[65]
4	IL-12 and IL-2	IL-12, IL-2	Dual cytokine therapy	Amplifies antitumor immune response	Cytokine release syndrome	[66]
5	IL-6 inhibition and chemotherapy	IL-6 antagonists	Cisplatin	Reduces chemotherapy resistance	Potential for exacerbating infection risks	[67]
6	IL-15 and NK cell adoptive transfer	IL-15	NK cell therapy	Enhances NK cell cytotoxicity	Cytokine-related toxicity	[50]
7	IL-10 blockade with PD-1 inhibitors	Anti-IL-10	PD-1/PD-L1 inhibitors	Reverses immune suppression in TME	Increased risk of autoimmune diseases	[68]
8	TGF-β inhibitors and VEGF inhibitors	TGF-β inhibitors	Anti-angiogenic agents	Reduces tumor vasculature	Potential for severe adverse vascular events	[69]
9	IL-1 blockades with radiotherapy	IL-1 antagonists	Radiation	Reduces tumor recurrence	Immunosuppressive side effects	[70]
10	IL-22 inhibition and checkpoint inhibitors	IL-22 inhibitors	PD-1/PD-L1 inhibitors	Overcomes immune evasion	Off-target effects on normal tissue	[71]
11	GM-CSF and cancer vaccines	GM-CSF	Peptide-based vaccines	Enhances vaccine efficacy	Inconsistent results across patient populations	[72]
12	IL-18 and chemotherapy	IL-18	Paclitaxel	Enhances chemotherapy-induced apoptosis	Severe cytokine-related toxicity	[73]
13	IL-2 and adoptive T cell therapy	IL-2	CAR-T therapy	Amplifies CAR-T cell efficacy	High toxicity and cytokine release syndrome	[74]
14	IFN-γ with oncolytic viruses	IFN-γ	Oncolytic viral therapy	Enhances viral-induced tumor cell death	High inflammatory response	[75]
15	TGF-β blockade with chemotherapy	TGF-β inhibitors	Cisplatin	Inhibits tumor progression and metastasis	Risk of systemic toxicity	[76]

IL-2: interleukin-2; IFN-α: interferon-alpha; TNF-α: tumor necrosis factor-alpha; NK: natural killer; TME: tumor microenvironment; TGF-β: transforming growth factor-beta

### Cytokines and ICIs: synergistic approaches

**Figure 1:**
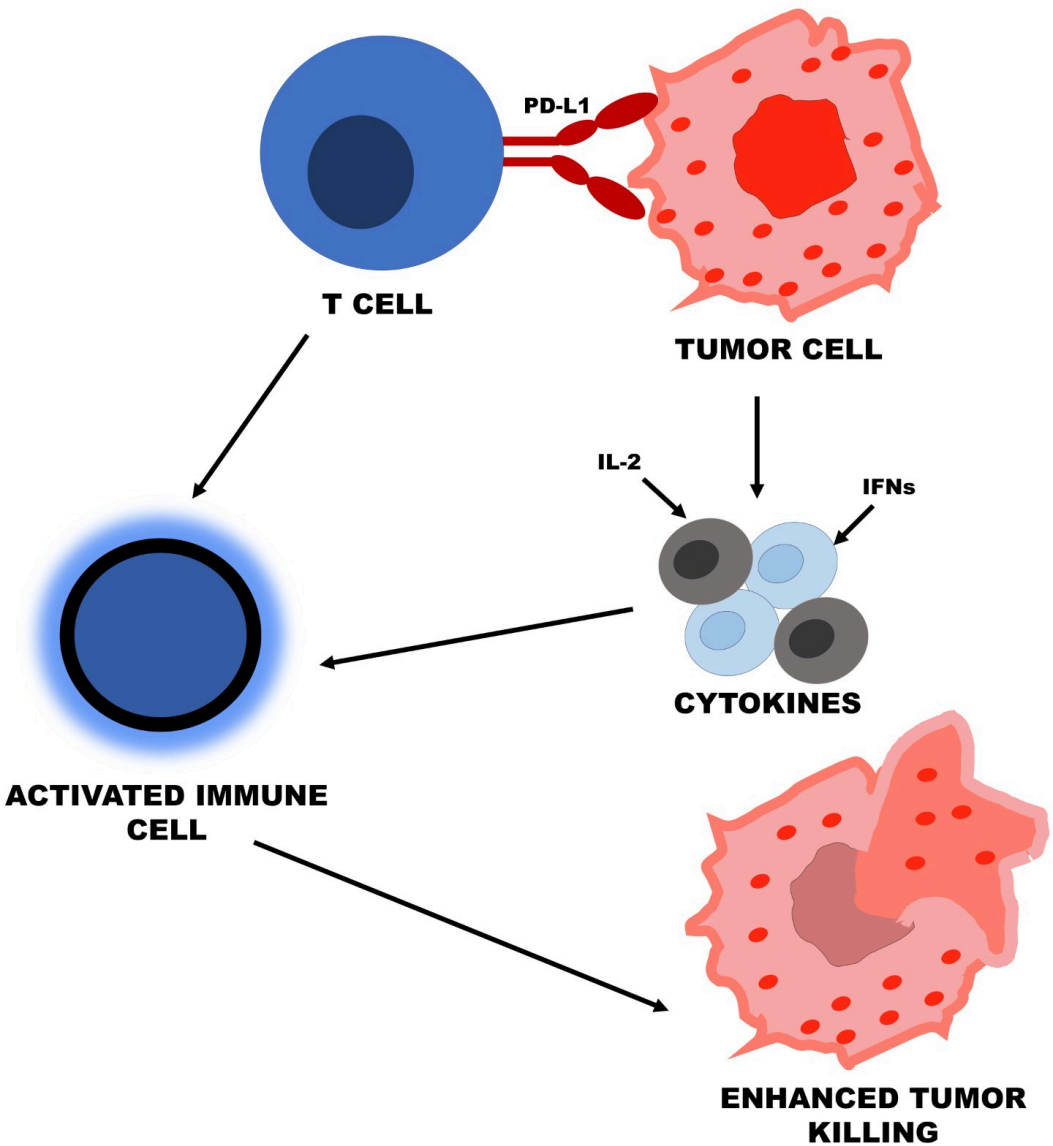
**Cytokines and immune checkpoint inhibitors**. IL-2: interleukin-2; IFNs: interferons

ICIs, including those targeting PD-1/PD-L1 or CTL antigen 4 (CTLA-4), have revolutionized cancer treatment: they are immunotherapy that increases the activity of T cells against tumors. They work, however, in only a minority of patients because these treatments are often blunted by mechanisms of immune resistance. This has added more activation of immune effector cells, like CTLs and NK cells, in the killing of tumor cells to cytokines, especially IL-2, since such cells play a critical role in antitumor cell responses. Results from the post-2020 studies indicated that IL-2 and anti-PD-1 therapy improve the antitumor immune response and slow down the progression of resistance by enhancing both innate and adaptive immunity. IFNs have also been shown to work with ICIs by improving the efficacy of the immune response against tumor cells [77]. Figure 1 illustrates the process of ICIs, such as PD-1, PD-L1, and CTLA-4, with cytokines, including IL-2, IFNs, and TNF-α. Immune cells activated in combination augment antitumor immunity and overcome the resistance experienced during gastric cancer therapy.

### Cytokine therapy with chemotherapy and radiotherapy

**Figure 2:**
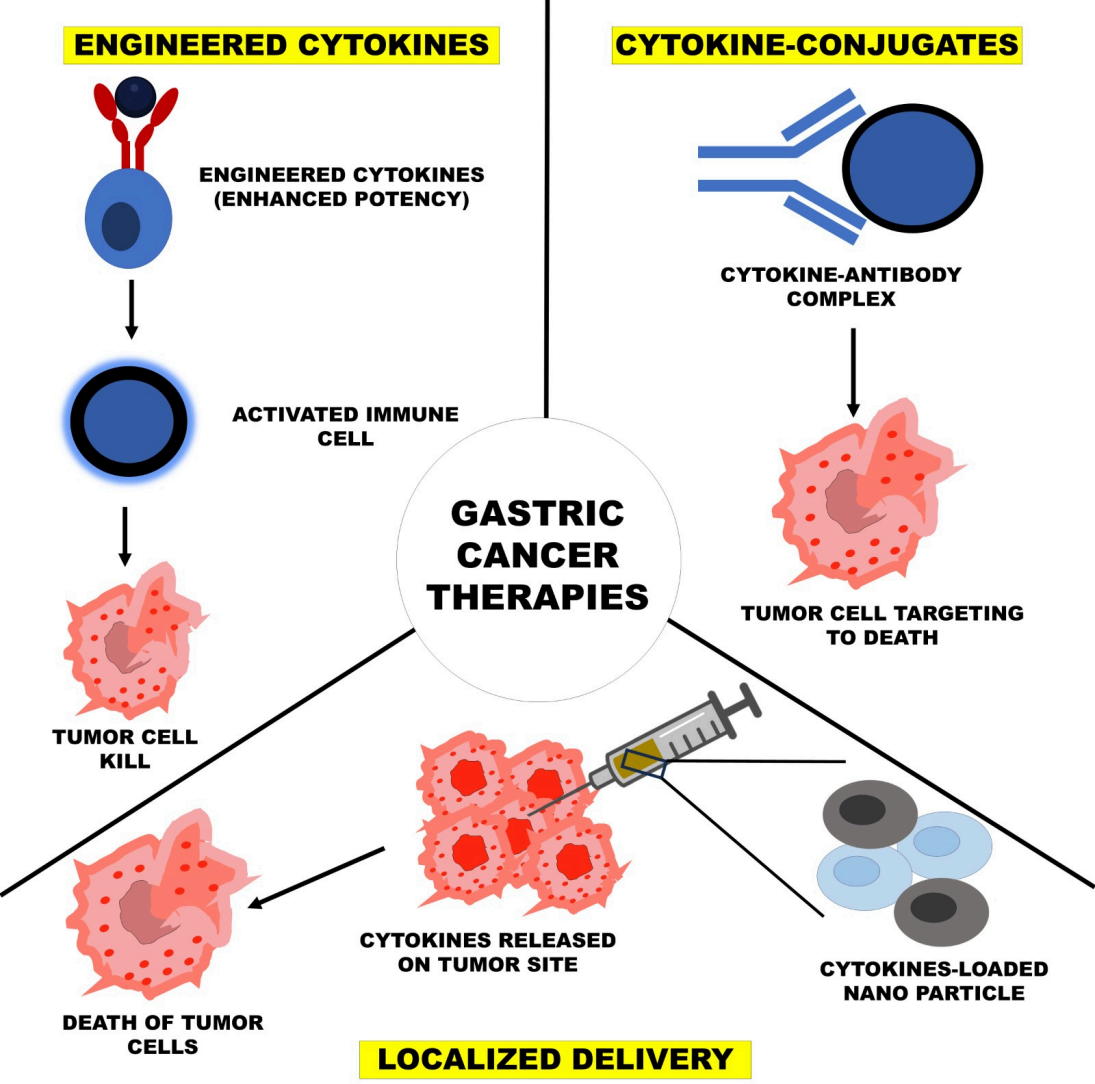
Cytokine-based therapies and targeted delivery mechanisms

Chemotherapy and radiotherapy are still the mainstay of cancer treatment but can generally dampen the immune system, thereby reducing their overall effectiveness. Cytokine therapy has been a significant thrust of recent research with traditional chemotherapy and radiotherapy. The application of these cytokines such as GM-CSF and TNF-α has been under research to negate the effect of immune suppression while stimulating an antitumor immune response, especially in synchronization with chemotherapy [8]. It has been shown through several studies that when administered together with radiotherapy, cytokines enhance local inflammation and subsequently increase immunogenic cell death characterized by decreased metastasis levels [78]. When radiotherapy leads to local damage, the tumor antigens are presented, and the cytokines enhance the immune response against such antigens, thereby making this an effective strategy in fighting cancer. Figure 2 shows three strategies of cytokine-based therapy, which include engineered cytokines for increased activation of the immune response, cytokine-conjugates that target the tumor for improved safety, and localized delivery systems that release cytokines directly at the tumor site. Each addresses the problem of improving therapeutic efficacy at the cost of reduced systemic toxicity and off-target effects.

### Overcoming immune resistance through cytokine modulation

Thus, immune resistance is one of the biggest hurdles in cancer therapy—one aspect of how tumors can react to intrinsic/induced changes that promote their survival by avoiding immune surveillance. Cytokines may then be used to modulate this immune resistance via pathways that enhance the activation of immune-activating pathways or inhibit immune-suppressive signals in TME. For instance, the addition of cytokine therapy to ICIs prevents the use of immune checkpoints by cancer, and cytokines like IL-12 and IL-15 have been found to increase the suppression of Tregs’ and MDSCs’ reduced immune response [79]. These immune cells are normally responsible for the repression of a suppressive TME that allows tumor evasions from immunodetection. Combination therapies targeting both immune suppression and activation pathways have shown promise in overtures both to resistance against cytokine therapy and against ICIs, demonstrated post 2020. Combining cytokines with ICIs, chemotherapy, and radiotherapy remains the promising strategy in being able to somehow improve the effectiveness of the treatment regimen for gastric cancer. Modulation of immune response that may be linked with decreased immune resistance may herald a new standard of care in the treatment of gastric cancer.

## Challenges and future directions

### Managing cytokine-related toxicity and side effects

These therapies hold promising prospects but still encounter several large-scale challenges. The most prominent among them is toxicity and side effects. Over-stimulation of the immune system can cause a condition called cytokine release syndrome (CRS), which is essentially a systemic inflammatory response leading to fever, fatigue, hypotension, and even multi-organ failure in severe cases. The most notorious examples of CRS were in case reports of IL-2 and CAR-T cell immunotherapy, which led to potentially lethal conditions due to overexuberant immune responses [80]. This balance is achieved very narrowly between getting the greatest possible response of the immune system against the cancer and avoiding off-target effects on normal tissues. Some recent approaches include dose optimization, the use of rationally engineered cytokines with higher specificity, and the combination of cytokine therapy with ICIs to minimize toxicity [81].

Moreover, researchers are working on local delivery systems for cytokines at the site of disease. Limiting systemic exposure and thereby less toxicity is one way to target cytokines to the TME. More and more recent developments in the form of cytokine-conjugates and liposome-based delivery systems improve the accuracy of cytokine therapies [82]. Such approaches shall have the maximum therapeutic potential with minimal adverse immune reactions.

### Biomarkers for predicting response to cytokine-based immunotherapy

Critical for the identification of reliable biomarkers is the task of predicting which patients could potentially be more responsive to cytokine-based immunotherapies. Among them, expression of PD-L1, tumor mutation burden (TMB), and the levels of circulating cytokines have been explored as predictors of response to ICIs, along with cytokine therapies [83]. Recent studies reveal that certain cytokines in TME, such as IL-10 and IFN-γ, may affect the treatment outcomes because the high levels of antitumor cytokines are related to better survival rates in patients with gastric cancer.

Advances in multi-omics technologies, such as transcriptomics, proteomics, and metabolomics, are also discovering new biomarkers that help predict the efficacy of therapy and tailor treatment strategies. For example, the integration of cytokine profiles with genetic and epigenetic markers can enable a clinician to narrow down a specific subset of patients who will best respond to that cytokine therapy, thus maximizing the outcome of treatment and minimizing potential toxicity that may be unfruitful [84].

### Emerging cytokine therapies and personalized medicine

Personalized medicine in cytokine-based immunotherapy is now highly pursued with an emphasis on tailoring individual treatments based on the unique characteristics of each patient’s tumor biology and immune status. Engineered cytokines, including modified IL-2 variants, have also shown promise in early-phase clinical trials for enhancing the therapeutic index by selectively activating immune cells that target tumor cells without harming normal tissues [30]. It also modulates the interactions of cytokines and their receptors for maximum efficacy with fewer side effects. For instance, pegylated cytokines are under development to enhance the half-life of cytokines and decrease immunogenicity [85].

The use of cytokine-based therapies in combination with other immunotherapies, such as adoptive T cell therapy and cancer vaccines, may complement the other to produce further synergistic benefits. Through manipulation of the immune microenvironment, these combination therapies should deliver inhibition of immune resistance mechanisms and enhance the potential of cytokine-based interventions. Results from ongoing clinical trials will provide promising information regarding the potential of such emerging therapies; hence, this next generation of personalized treatments for gastric cancer [86].

## Conclusions

Immunotherapy based on cytokines aimed at gastric carcinoma represents a new and promising route that should accelerate the process of developing treatment concepts. This review discusses such issues as cytokines being either tumor growth promoters or inhibitors. For instance, it has been established that pro-inflammatory cytokines such as IL-2, IFNs, and TNF-α are possessed with a greater repute for cell-mediated immunity activation, for example, through CTL and NK cell activation which leads to tumor growth inhibition. Yet, their therapeutic application in practice is far from free of difficulties. On the one hand, a strong pleiotropic activity of cytokines may distort the extent of systemic inflammation or toxicity and sometimes even suppress immunity, which makes much greater obstacles to broader clinical application.

Advances in engineered cytokines, cytokine-conjugates, and localized delivery systems of cytokines are bright hope over these challenges. They target increasing the therapeutic index by attempting to enhance the killing capacity of tumor cells by reducing the off-target toxicities. Indeed, combining ICIs, chemotherapy, or radiotherapy, cytokine-based immunotherapies can work synergistically to combat immune resistance and improve the outcome of treatment.

The TME plays an important role in influencing immune responses, where immunosuppressive cytokines like IL-10 and TGF-β drive mechanisms for immune evasion. Therapeutic targeting of these suppressive pathways in combination with cytokine-based treatments may offer a combinatory approach to reconstitute antitumor immunity.

Despite such huge promises from cytokine-based therapies, more research is needed for handling toxicities and to develop reliable biomarkers that can provide a predictive quotient for a patient’s response. Emerging cytokine therapies and personalized medicine approaches based on immune profiles for individual patients will determine the future of gastric cancer treatment. Clinical trials already in progress have provided comprehensive results for the definition of place for cytokine-based therapies in management alone and transformed the landscape of cancer immunotherapy.

## Abbreviations

CTLs: cytotoxic T lymphocytes
ICIs: immune checkpoint inhibitors
IFNs: interferons
IL-2: interleukin-2
NK: natural killer
TAM: tumor-associated macrophage
TGF-β: transforming growth factor-beta
TME: tumor microenvironment
TNF-α: tumor necrosis factor-alpha
Treg: regulatory T cell

## Declarations

### Copyright

© The Author(s) 2025.

## References

[1] Jamil D, Palaniappan S, Lokman A, Naseem M, Zia SS (2021). Diagnosis of Gastric Cancer Using Machine Learning Techniques in Healthcare Sector: A Survey. Informatica.

[2] Ramachandran S, Verma AK, Dev K, Goyal Y, Bhatt D, Alsahli MA (2021). Role of Cytokines and Chemokines in NSCLC Immune Navigation and Proliferation. Oxid Med Cell Longev.

[3] Fang J, Lu Y, Zheng J, Jiang X, Shen H, Shang X (2023). Exploring the crosstalk between endothelial cells, immune cells, and immune checkpoints in the tumor microenvironment: new insights and therapeutic implications. Cell Death Dis.

[4] Tahmasebi S, Alimohammadi M, Khorasani S, Rezaei N, Rezaei N (2022). Pro-tumorigenic and Anti-tumorigenic Roles of Pro-inflammatory Cytokines in Cancer. Handbook of Cancer and Immunology.

[5] Abdul-Rahman T, Ghosh S, Badar SM, Nazir A, Bamigbade GB, Aji N (2024). The paradoxical role of cytokines and chemokines at the tumor microenvironment: a comprehensive review. Eur J Med Res.

[6] Chen C, Wang Z, Ding Y, Qin Y (2023). Tumor microenvironment-mediated immune evasion in hepatocellular carcinoma. Front Immunol.

[7] Majidpoor J, Mortezaee K (2021). Interleukin-2 therapy of cancer-clinical perspectives. Int Immunopharmacol.

[8] MacDonald A, Wu TC, Hung CF (2021). Interleukin 2-Based Fusion Proteins for the Treatment of Cancer. J Immunol Res.

[9] Deckers J, Anbergen T, Hokke AM, de Dreu A, Schrijver DP, de Bruin K (2023). Engineering cytokine therapeutics. Nat Rev Bioeng.

[10] Rybchenko VS, Aliev TK, Panina AA, Kirpichnikov MP, Dolgikh DA (2023). Targeted Cytokine Delivery for Cancer Treatment: Engineering and Biological Effects. Pharmaceutics.

[11] Yu S, Wang Y, He P, Shao B, Liu F, Xiang Z (2022). Effective Combinations of Immunotherapy and Radiotherapy for Cancer Treatment. Front Oncol.

[12] Liang JL, Luo GF, Chen WH, Zhang XZ (2021). Recent Advances in Engineered Materials for Immunotherapy-Involved Combination Cancer Therapy. Adv Mater.

[13] Mallick R, Basak S, Chowdhury P, Bhowmik P, Das RK, Banerjee A (2025). Targeting Cytokine-Mediated Inflammation in Brain Disorders: Developing New Treatment Strategies. Pharmaceuticals (Basel).

[14] Bi Q, Wu JY, Qiu XM, Zhang JD, Sun ZJ, Wang W (2022). Tumor-Associated Inflammation: The Tumor-Promoting Immunity in the Early Stages of Tumorigenesis. J Immunol Res.

[15] Ghemrawi R, Abuamer L, Kremesh S, Hussien G, Ahmed R, Mousa W (2024). Revolutionizing Cancer Treatment: Recent Advances in Immunotherapy. Biomedicines.

[16] Bandara S, Raveendran S (2025). Current Landscape and Future Directions in Cancer Immunotherapy: Therapies, Trials, and Challenges. Cancers (Basel).

[17] Yang YL, Yang F, Huang ZQ, Li YY, Shi HY, Sun Q (2023). T cells, NK cells, and tumor-associated macrophages in cancer immunotherapy and the current state of the art of drug delivery systems. Front Immunol.

[18] Fenton SE, Saleiro D, Platanias LC (2021). Type I and II Interferons in the Anti-Tumor Immune Response. Cancers (Basel).

[19] Zhu Q, Huang X, Deng B, Guan L, Zhou H, Shi B (2024). Tumor micro-environment induced TRAIL secretion from engineered macrophages for anti-tumor therapy. Cell Immunol.

[20] Batchu RB, Gruzdyn OV, Kolli BK, Dachepalli R, Umar PS, Rai SK, Birbrair A (2021). IL-10 Signaling in the Tumor Microenvironment of Ovarian Cancer. Tumor Microenvironment : The Role of Interleukins – Part B.

[21] Singh S, Gouri V, Samant M (2023). TGF-β in correlation with tumor progression, immunosuppression and targeted therapy in colorectal cancer. Med Oncol.

[22] Jiang J, Zhu F, Zhang H, Sun T, Fu F, Chen X (2022). Luteolin suppresses the growth of colon cancer cells by inhibiting the IL-6/STAT3 signaling pathway. J Gastrointest Oncol.

[23] Rose-John S (2021). Therapeutic targeting of IL-6 trans-signaling. Cytokine.

[24] Glassman CR, Mathiharan YK, Jude KM, Su L, Panova O, Lupardus PJ (2021). Structural basis for IL-12 and IL-23 receptor sharing reveals a gateway for shaping actions on T versus NK cells. Cell.

[25] Hunzeker ZE, Zhao L, Kim AM, Parker JM, Zhu Z, Xiao H (2024). The role of IL-22 in cancer. Med Oncol.

[26] Morand S, Devanaboyina M, Fung C, Royfman R, Filipiak L, Stanbery L (2021). GM-CSF: Anti-Cancer Immune Response and Therapeutic Application. J Vaccines Vaccin.

[27] Zhang S, Zhao J, Bai X, Handley M, Shan F (2021). Biological effects of IL-15 on immune cells and its potential for the treatment of cancer. Int Immunopharmacol.

[28] Tan Y, Wang M, Zhang Y, Ge S, Zhong F, Xia G (2021). Tumor-Associated Macrophages: A Potential Target for Cancer Therapy. Front Oncol.

[29] Somm E, Jornayvaz FR (2022). Interleukin-18 in metabolism: From mice physiology to human diseases. Front Endocrinol (Lausanne).

[30] Balkhi S, Bilato G, De Lerma Barbaro A, Orecchia P, Poggi A, Mortara L (2025). Efficacy of Anti-Cancer Immune Responses Elicited Using Tumor-Targeted IL-2 Cytokine and Its Derivatives in Combined Preclinical Therapies. Vaccines (Basel).

[31] Labani-Motlagh A, Ashja-Mahdavi M, Loskog A (2020). The Tumor Microenvironment: A Milieu Hindering and Obstructing Antitumor Immune Responses. Front Immunol.

[32] Blaauboer A, Sideras K, van Eijck CHJ, Hofland LJ (2021). Type I interferons in pancreatic cancer and development of new therapeutic approaches. Crit Rev Oncol Hematol.

[33] Fang F, Zhang T, Li Q, Chen X, Jiang F, Shen X (2022). The tumor immune-microenvironment in gastric cancer. Tumori.

[34] Zhang X, Wu J (2021). LINC00665 promotes cell proliferation, invasion, and metastasis by activating the TGF-β pathway in gastric cancer. Pathol Res Pract.

[35] Yi M, Li T, Niu M, Zhang H, Wu Y, Wu K (2024). Targeting cytokine and chemokine signaling pathways for cancer therapy. Signal Transduct Target Ther.

[36] Overwijk WW, Tagliaferri MA, Zalevsky J (2021). Engineering IL-2 to Give New Life to T Cell Immunotherapy. Annu Rev Med.

[37] Borówka M, Łącki-Zynzeling S, Nicze M, Kozak S, Chudek J (2022). Adverse Renal Effects of Anticancer Immunotherapy: A Review. Cancers (Basel).

[38] Ding H, Wang G, Yu Z, Sun H, Wang L (2022). Role of interferon-gamma (IFN-γ) and IFN-γ receptor 1/2 (IFNγR1/2) in regulation of immunity, infection, and cancer development: IFN-γ-dependent or independent pathway. Biomed Pharmacother.

[39] Appleton E, Hassan J, Chan Wah Hak C, Sivamanoharan N, Wilkins A, Samson A (2021). Kickstarting Immunity in Cold Tumours: Localised Tumour Therapy Combinations With Immune Checkpoint Blockade. Front Immunol.

[40] Hu M, Huang L (2022). Strategies targeting tumor immune and stromal microenvironment and their clinical relevance. Adv Drug Deliv Rev.

[41] Stenger TD, Miller JS (2024). Therapeutic approaches to enhance natural killer cell cytotoxicity. Front Immunol.

[42] Rallis KS, Corrigan AE, Dadah H, George AM, Keshwara SM, Sideris M (2021). Cytokine-based Cancer Immunotherapy: Challenges and Opportunities for IL-10. Anticancer Res.

[43] Dakhel S, Lizak C, Matasci M, Mock J, Villa A, Neri D (2021). An Attenuated Targeted-TNF Localizes to Tumors In Vivo and Regains Activity at the Site of Disease. Int J Mol Sci.

[44] Nagata K, Nishiyama C (2021). IL-10 in Mast Cell-Mediated Immune Responses: Anti-Inflammatory and Proinflammatory Roles. Int J Mol Sci.

[45] Zhao H, Wei J, Sun J (2020). Roles of TGF-β signaling pathway in tumor microenvirionment and cancer therapy. Int Immunopharmacol.

[46] Li R, Wen A, Lin J (2020). Pro-Inflammatory Cytokines in the Formation of the Pre-Metastatic Niche. Cancers (Basel).

[47] Xin Z, Qu S, Qu Y, Xu Y, Liu R, Sun D (2024). Emerging IL-12-based nanomedicine for cancer therapy. Nano Today.

[48] De Benedetti F, Prencipe G, Bracaglia C, Marasco E, Grom AA (2021). Targeting interferon-γ in hyperinflammation: opportunities and challenges. Nat Rev Rheumatol.

[49] Di Gioacchino M, Della Valle L, Allegra A, Pioggia G, Gangemi S (2022). AllergoOncology: Role of immune cells and immune proteins. Clin Transl Allergy.

[50] Zhou Y, Husman T, Cen X, Tsao T, Brown J, Bajpai A (2022). Interleukin 15 in Cell-Based Cancer Immunotherapy. Int J Mol Sci.

[51] Zhu YH, Zheng JH, Jia QY, Duan ZH, Yao HF, Yang J (2023). Immunosuppression, immune escape, and immunotherapy in pancreatic cancer: focused on the tumor microenvironment. Cell Oncol (Dordr).

[52] Mortara L, Balza E, Bruno A, Poggi A, Orecchia P, Carnemolla B (2018). Anti-cancer Therapies Employing IL-2 Cytokine Tumor Targeting: Contribution of Innate, Adaptive and Immunosuppressive Cells in the Anti-tumor Efficacy. Front Immunol.

[53] Cui W, Hull L, Zizzo A, Wang L, Lin B, Zhai M (2023). Pharmacokinetic Study of rhIL-18BP and Its Effect on Radiation-Induced Cytokine Changes in Mouse Serum and Intestine. Toxics.

[54] Brusini R, Varna M, Couvreur P (2020). Advanced nanomedicines for the treatment of inflammatory diseases. Adv Drug Deliv Rev.

[55] Gottschlich A, Endres S, Kobold S (2021). Therapeutic Strategies for Targeting IL-1 in Cancer. Cancers (Basel).

[56] Metanat Y, Viktor P, Amajd A, Kaur I, Hamed AM, Abed Al-Abadi NK (2024). The paths toward non-viral CAR-T cell manufacturing: A comprehensive review of state-of-the-art methods. Life Sci.

[57] Yasasve M, Manjusha M, Saravanan M (2022). Polymorphism in pro-inflammatory cytokines and their genetic susceptibility towards oral precancerous lesions and oral cancer. Oral Oncol.

[58] Fu Y, Tang R, Zhao X (2023). Engineering cytokines for cancer immunotherapy: a systematic review. Front Immunol.

[59] Brouillard A, Deshpande N, Kulkarni AA (2021). Engineered Multifunctional Nano- and Biological Materials for Cancer Immunotherapy. Adv Healthc Mater.

[60] Saadh MJ, Rasulova I, Khalil M, Farahim F, Sârbu I, Ciongradi CI (2024). Natural killer cell-mediated immune surveillance in cancer: Role of tumor microenvironment. Pathol Res Pract.

[61] Wang S, Cheng K, Chen K, Xu C, Ma P, Dang G (2022). Nanoparticle-based medicines in clinical cancer therapy. Nano Today.

[62] Saadh MJ, Baher H, Li Y, Chaitanya M, Arias-Gonzáles JL, Allela OQB (2023). The bioengineered and multifunctional nanoparticles in pancreatic cancer therapy: Bioresponisive nanostructures, phototherapy and targeted drug delivery. Environ Res.

[63] Okiyama N, Tanaka R (2022). Immune-related adverse events in various organs caused by immune checkpoint inhibitors. Allergol Int.

[64] Wang Z, Pang S, Liu X, Dong Z, Tian Y, Ashrafizadeh M (2024). Chitosan- and hyaluronic acid-based nanoarchitectures in phototherapy: Combination cancer chemotherapy, immunotherapy and gene therapy. Int J Biol Macromol.

[65] Zhu S, Wang Y, Tang J, Cao M (2022). Radiotherapy induced immunogenic cell death by remodeling tumor immune microenvironment. Front Immunol.

[66] Mirlekar B, Pylayeva-Gupta Y (2021). IL-12 Family Cytokines in Cancer and Immunotherapy. Cancers (Basel).

[67] Domingo IK, Latif A, Bhavsar AP (2022). Pro-Inflammatory Signalling PRRopels Cisplatin-Induced Toxicity. Int J Mol Sci.

[68] Jimbu L, Mesaros O, Neaga A, Nanut AM, Tomuleasa C, Dima D (2021). The Potential Advantage of Targeting Both PD-L1/PD-L2/PD-1 and IL-10-IL-10R Pathways in Acute Myeloid Leukemia. Pharmaceuticals (Basel).

[69] Elebiyo TC, Rotimi D, Evbuomwan IO, Maimako RF, Iyobhebhe M, Ojo OA (2022). Reassessing vascular endothelial growth factor (VEGF) in anti-angiogenic cancer therapy. Cancer Treat Res Commun.

[70] Donlon NE, Power R, Hayes C, Reynolds JV, Lysaght J (2021). Radiotherapy, immunotherapy, and the tumour microenvironment: Turning an immunosuppressive milieu into a therapeutic opportunity. Cancer Lett.

[71] Park K, Veena MS, Shin DS (2022). Key Players of the Immunosuppressive Tumor Microenvironment and Emerging Therapeutic Strategies. Front Cell Dev Biol.

[72] Zhou Y (2023). HER2/neu-based vaccination with li-Key hybrid, GM-CSF immunoadjuvant and trastuzumab as a potent triple-negative breast cancer treatment. J Cancer Res Clin Oncol.

[73] Braumüller H, Mauerer B, Andris J, Berlin C, Wieder T, Kesselring R (2023). The Cytokine Network in Colorectal Cancer: Implications for New Treatment Strategies. Cells.

[74] Balagopal S, Sasaki K, Kaur P, Nikolaidi M, Ishihara J (2022). Emerging approaches for preventing cytokine release syndrome in CAR-T cell therapy. J Mater Chem B.

[75] Ehrlich M, Bacharach E (2021). Oncolytic Virotherapy: The Cancer Cell Side. Cancers (Basel).

[76] Chen J, Ding ZY, Li S, Liu S, Xiao C, Li Z (2021). Targeting transforming growth factor-β signaling for enhanced cancer chemotherapy. Theranostics.

[77] Kumaravel JJ, Anbalagan G, Selvamani M, Elangovan D, Subramanian B (2024). An exciting new approach to cancer treatment: Cryo-immune engineering and its mechanism. Oral Oncol Rep.

[78] Zhang Z, Liu X, Chen D, Yu J (2022). Radiotherapy combined with immunotherapy: the dawn of cancer treatment. Signal Transduct Target Ther.

[79] Kuske M, Haist M, Jung T, Grabbe S, Bros M (2022). Immunomodulatory Properties of Immune Checkpoint Inhibitors—More than Boosting T-Cell Responses?. Cancers (Basel).

[80] Kannan B, Jayaseelan VP, Arumugam P (2023). Immunotherapy for oral cancer treatment through targeting of IDO1 and its pathway. J Stomatol Oral Maxillofac Surg.

[81] Zheng X, Wu Y, Bi J, Huang Y, Cheng Y, Li Y (2022). The use of supercytokines, immunocytokines, engager cytokines, and other synthetic cytokines in immunotherapy. Cell Mol Immunol.

[82] Senthil R, Raghunandhakumar S (2024). Helping anti-cancer activity of cetuximab (Erbitux) by monoclonal antibody conjugated nanocurcumin - In vitro studies on human oral squamous carcinoma cell line (OECM). Oral Oncol Rep.

[83] Ji S, Chen H, Yang K, Zhang G, Mao B, Hu Y (2020). Peripheral cytokine levels as predictive biomarkers of benefit from immune checkpoint inhibitors in cancer therapy. Biomed Pharmacother.

[84] Brands X, Haak BW, Klarenbeek AM, Butler J, Uhel F, Qin W (2021). An epigenetic and transcriptomic signature of immune tolerance in human monocytes through multi-omics integration. Genome Med.

[85] Xue D, Hsu E, Fu YX, Peng H (2021). Next-generation cytokines for cancer immunotherapy. Antib Ther.

[86] Catanese S, Lordick F (2021). Targeted and immunotherapy in the era of personalised gastric cancer treatment. Best Pract Res Clin Gastroenterol.

